# Global, regional, national burden and gender disparity of cataract: findings from the global burden of disease study 2019

**DOI:** 10.1186/s12889-022-14491-0

**Published:** 2022-11-12

**Authors:** Rui Fang, Yang-Fan Yu, En-Jie Li, Ning-Xin Lv, Zhao-Chuan Liu, Hong-Gang Zhou, Xu-Dong Song

**Affiliations:** 1 Beijing Tongren Hospital, Beijing, 100730 China; 2 Capital Medical University, Beijing, 100730 China; 3 Beijing Tongren Eye Center, Beijing, 100730 China; 4 Beijing Ophthalmology&Visual Sciences Key Lab, Beijing, 100730 China; 5 The State Key Laboratory of Medicinal Chemical Biology, College of Pharmacy and Key Laboratory of Molecular Drug Research, Nan kai University, Tianjin, China

**Keywords:** Cataract, Global burden of disease, Vision loss, Disability-adjusted life year

## Abstract

**Background:**

To evaluate the global burden of cataracts by year, age, region, gender, and socioeconomic status using disability-adjusted life years (DALYs) and prevalence from the Global Burden of Disease (GBD) study 2019.

**Methods:**

Global, regional, or national DALY numbers, crude DALY rates, and age-standardized DALY rates caused by cataracts, by year, age, and gender, were obtained from the Global Burden of Disease Study 2019. Socio-demographic Index (SDI) as a comprehensive indicator of the national or regional development status of GBD countries in 2019 was obtained from the GBD official website. Kruskal-Wallis test, linear regression, and Pearson correlation analysis were performed to explore the associations between the health burden with socioeconomic levels, Wilcoxon Signed-Rank Test was used to investigate the gender disparity.

**Results:**

From 1990 to 2019, global DALY numbers caused by cataracts rose by 91.2%, crude rates increased by 32.2%, while age-standardized rates fell by 11.0%. Globally, age-standardized prevalence and DALYs rates of cataracts peaked in 2017 and 2000, with the prevalence rate of 1283.53 [95% uncertainty interval (UI) 1134.46–1442.93] and DALYs rate of 94.52 (95% UI 67.09–127.24) per 100,000 population, respectively. The burden was expected to decrease to 1232.33 (95% UI 942.33–1522.33) and 91.52 (95% UI 87.11–95.94) by 2050. Southeast Asia had the highest blindness rate caused by cataracts in terms of age-standardized DALY rates (99.87, 95% UI: 67.18–144.25) in 2019. Gender disparity has existed since 1990, with the female being more heavily impacted. This pattern remained with aging among different stages of vision impairments and varied through GBD super regions. Gender difference (females minus males) of age-standardized DALYs (equation: Y = -53.2*X + 50.0, *P* < 0.001) and prevalence rates (equation: Y = − 492.8*X + 521.6, *P* < 0.001) was negatively correlated with SDI in linear regression.

**Conclusion:**

The global health of cataracts is improving but the steady growth in crude DALY rates suggested that health progress does not mean fewer demands for cataracts. Globally, older age, females, and lower socioeconomic status are associated with higher cataract burden. The findings of this study highlight the importance to make gender-sensitive health policies to manage global vision loss caused by cataracts, especially in low SDI regions.

**Supplementary Information:**

The online version contains supplementary material available at 10.1186/s12889-022-14491-0.

## Introduction

Cataract, defined as loss of lens transparency, causes alteration of refractive properties and elevated light scattering, resulting in hazy vision or blindness [1]. Cataract is one of the leading causes of blindness worldwide [2, 3]. The prevalence of cataracts increases with age, ranging from 3.9% among 55–64 years to 92.6% among those 80 years and older [4]. In 2010, there were 10.8 million cataract blind people [5], this number is expected to increase to 40 million in 2025 with the aging of the world’s populations and greater life expectancies [6]. The impact of cataracts on visual loss, especially in an elderly population, exacerbates the risk of dementia [7], increases the likelihood of falls and road traffic crashes [8, 9], can markedly affect the quality of an individual’s life and ultimately leads to higher mortality [10].

As yet, there are no preventative or therapeutic drugs against cataracts have been approved, leaving surgery as the only effective treatment option [11]. Due to refinements in modern cataract Surgical techniques, the procedure is considered to be relatively safe and can improve the visual function of patients well [12]. Research has found that timely and equitable access to cataract surgery can prevent fall-related injuries and support healthy aging [8, 9]. Some studies also suggest that cataract extraction is associated with a lower risk of developing dementia among older adults [7]. Given our globally aging population, the number of people affected with cataracts is predicted to increase worldwide, especially in low-income nations with limited access to surgery [13]. The management of cataracts will become a socioeconomic challenge since the social and economic costs of cataracts are quite staggering and the demand for cataract surgery far exceeds limited public health resources.

Despite the efficacy and advantage of cataract surgery, it is not equally accessible to the world’s population [14, 15]. Especially in developing countries, people might not have the necessary financial means or access to a surgeon who can operate cataract surgeries. Hence, most cataract around the world remains untreated. Disease burden, cataract surgical rate/coverage, and human resources are endorsed as the national indicator for monitoring eye services by the World Health Assembly [16]. The health burden of disease can be quantified using disability-adjusted life years (DALYs). DALYs are the sum of years lived with disability (YLDs) and years of life lost (YLLs) owing to premature death [17]. To better evaluate the global burden of cataracts, we use prevalence and DALYs from the GBD 2019 study as the main measurements to make comparisons across the year, age, region, gender, and socioeconomic status. We aimed to raise the attention of the public to prompt the treatment of cataracts and provide a reference for health policymaking.

## Methods

### Overview

The GBD study estimates incidence, prevalence, mortality, YLLs, YLDs, and DALYs due to different diseases and injuries, based on the data extracted from censuses, household surveys, civil registration and vital statistics, disease registries, health service use, air pollution monitors and other sources [17]. Uncertainty intervals (UIs) were generated for every metric using the 25th and 975th ordered 1000 draw values of the posterior distribution. Ethics approval and informed consent were not required for this study because of public accessibility to the data.

### Data extraction

Detailed methods for estimating DALYs have been mentioned in related publications [17, 18]. According to the GBD 2019 study, DALYs estimates for cataracts were equal to YLDs [17]. To avoid confusion, DALYs are used uniformly below.

Interested data regarding cataracts were collected from the Global Health Data Exchange (http://ghdx.healthdata.org/gbd-results-tool), including the following: (1) global total and age- and gender-specific data of prevalence and DALYs, as absolute number and age-standardized rates (per 100,000 population) from 1990 to 2019; (2) global total and gender-specific data of prevalence and DALYs from 1990 to 2019, as crude rates (per 100,000 population); (3) GBD super regions total and gender-specific prevalence and DALYs data in 1990 and 2019, as age-standardized rates of causes attribute to vision loss in 1990 and 2019; (4) World Health Orgnization (WHO) regions total and gender-specific prevalence and DALYs, as absolute number and age-standardized rates from 1990 to 2019 and (5)Socio-demographic Index (SDI) of GBD countries in 2019.

Moderate and severe vision impairment (MSVI) is defined as visual acuity (VA) worse than 6/18, but equal to or better than 3/60, while the blindness definition was VA < 3/60 or visual field around central fixation < 10%, based on Snellen charts in meters. Total vision loss equaled the sum of different stages of vision loss [10].

### Socioeconomic status

The SDI is a composite indicator of income per capita, years of schooling, and fertility rate in females younger than 25 years [18]. The SDI varies from 0 to 1, with a higher value implicating better socioeconomic development: high SDI countries (SDI > 0.81), high-middle SDI countries (0.70 < SDI ≤ 0.81), middle SDI countries(0.60 < SDI ≤ 0.70), low-middle SDI countries (0.46 < SDI ≤ 0.60) and low SDI countries (SDI ≤ 0.46).

### Forecasting cataract burden beyond 2019

Auto-Regressive Integrated Moving Average (ARIMA) model is a popular and widely used statistical method for time series prediction in epidemiological studies [19]. ARIMA model was performed in R Statistical Software (version 4.1.2) with forecast (version 8.16) and tseries (version 0.10–51) packages to forecast the health burden caused by cataracts from 2018 towards 2050 in terms of age-standardized rates of DALY and prevalence. ARIMA is specified by three main component parameters known as p, d, and q. P stands for autoregression, represents the number of lag observations in the model; d stands for integrated, represents the number of times input raw data are differenced and q stands for moving average, represents the size of moving average window applied to lagged observations. We established an ARIMA model to make the prediction and then tested the model.

### Statistical analyses

Age-standardized rates and crude rates of DALY and prevalence were expressed as the number per 100,000 population with 95% UIs. Comparisons of gender differences in national DALY numbers and crude DALY rates for each age group were performed by Wilcoxon signed-rank test. The differences in age-standardized DALY rates among five SDI-based country groups were explored by the Kruskal-Wallis test, Pearson correlation analyses, and linear regression analyses were performed to explore the relationship between gender difference in age-standardized DALY rates and prevalence rates with SDI.

All statistical analyses were performed using R Statistical Software (version 4.1.2; R Foundation for Statistical Computing, Vienna, Austria). *P* value less than 0.05 was considered statistically significant.

## Results

### Global trends in DALY and prevalence of cataracts

**Fig. 1:**
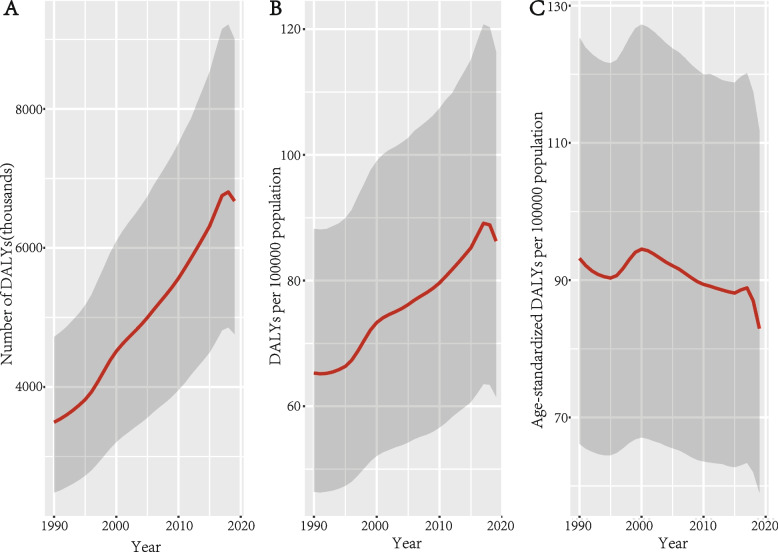
Trends in global burden of cataracts in terms of DALY numbers (**A**), crude DALY rates (**B**), and age-standardized DALY rates (**C**), from 1990 to 2019. Shaded areas represent 95% uncertainty intervals. DALYs = disability-adjusted life years

The absolute numbers of DALYs and prevalence caused by cataracts witnessed ascending trends over the past decades, both peaked in 2018 at 6.80 million (95%UI:4.81–9.21) and 98.41 million (95%UI:86.94–110.98) respectively, but fall simultaneously in 2019 (Fig. 1A). After accounting for the growing population, the crude DALY rates increased by 32.2% from 65.28 (95% UI: 46.39–88.22) in 1990 to 86.29 (95% UI: 61.53–116.40) in 2019 and the crude prevalence rates rose by 58.5% from 791.36 (95% UI: 705.23–890.03) to 1253.93 (95% UI: 1103.34–1417.73) in 2019 (Fig. 1B). After adjusting for population size and age structure, the age-standardized DALY rates fell by 11.0% from 93.17 (95% UI: 66.14–125.32) in 1990 to 82.94 (95% UI: 59.06–111.75) in 2019 (Fig. 1C). However, the age-standardized prevalence rates rose slightly from 1150.56 (95% UI: 1027.31–1287.40) to 1207.89 (95% UI: 1065.04–1361.26) (Fig. S1).

### Cataract burden stratified by age and gender

**Fig. 2:**
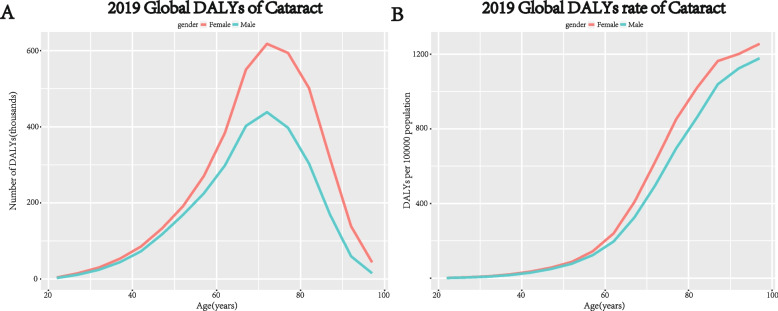
Global burden of cataracts in terms of DALY numbers (**A**), crude DALY rates (**B**) by age and gender in 2019. DALYs = disability-adjusted life years

GBD Study 2019 started to capture DALYs due to cataracts for persons aged 20–24 years old, the DALY numbers and crude DALY rates stratified by age and gender were available for 204 countries. Wilcoxon Signed-Rank Test showed significant gender differences in global DALY numbers and crude DALY rates in different age groups (*p* < 0.05), global DALYs numbers and DALY rates were higher in older females than in males of the same age. The changes in DALY numbers by age were similar in both sexes, remaining steady growth and reaching two peaks in the age range of 70 to 74 years. The gender inequality of DALYs increased with age, while peaked in the 80–84 age group, with DALYs of 0.50 (95% UI: 0.35–0.68) million among women versus 0.30 (95% UI: 0.21–0.42) million among men (Fig. 2A). The crude DALY rates by age in both genders have a similar trend, increasing slowly under 50 years of age and rapidly above 50 years of age (Fig. 2B).

### Cataract burden by socioeconomic status and region

**Fig. 3:**
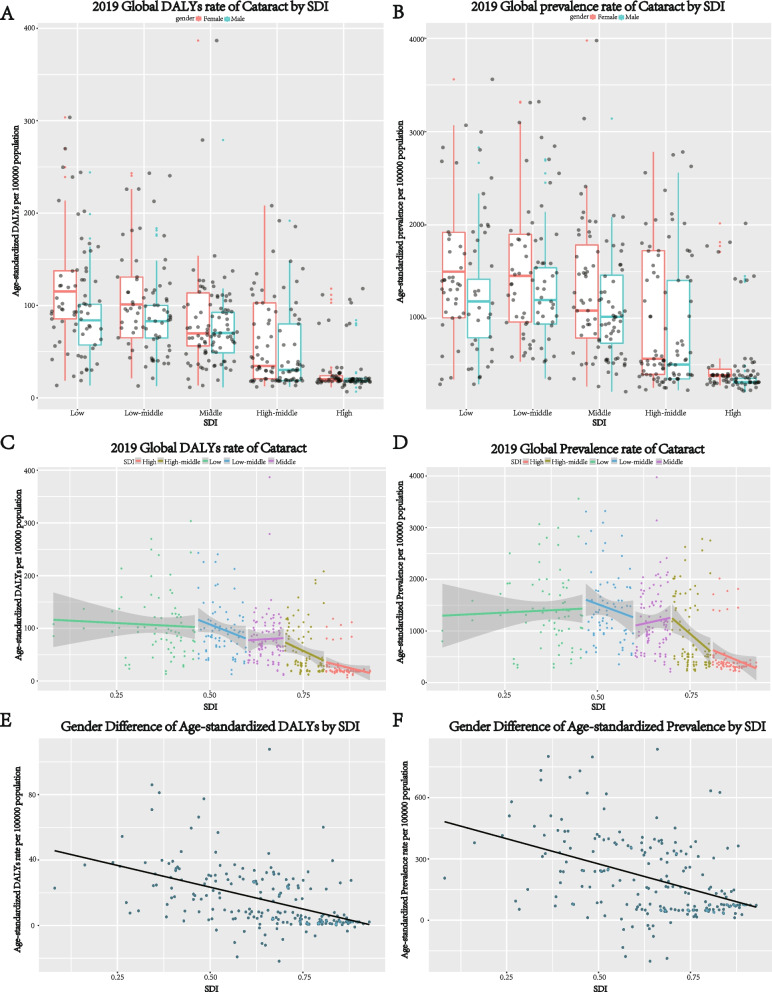
Health burden of cataracts in SDI regions in 2019. Gender-specific burden in terms of age-standardized DALY rates (**A**), and age-standardized prevalence rates (**B**) in 204 countries. Age- standardized DALY rates (**C**), and age-standardized prevalence rates (**D**) in different SDI regions. Association between gender difference of age-standardized DALY rates (**E**), and age-standardized prevalence rates (**F**) with SDI. Shaded areas represent 95% uncertainty intervals. SDI = socio-demographic index; DALYs = disability-adjusted life years

SDI data in 2019 were available for 204 countries and were classified into five groups, including 39 high-SDI, 45 high-middle-SDI, 47 medium-SDI, 37 low-middle-SDI and 36 low-SDI countries and territories. Kruskal-Wallis tests indicated that age-standardized DALY rates differed significantly among countries with different SDI regions in 2019 (χ2 (4) = 151.81, *p* < 0.001). At the same time, there was strong difference of age-standardized prevalence rates among countries in different SDI regions in 2019 (χ2(4) = 129.76, *p* < 0.001). Multiple comparisons using Bonferroni Correction revealed higher both age-standardized rates of DALY and prevalence in lower HDI countries. The medians (interquartile ranges) of age-standardized DALY rates in low, low-middle, middle,high-middle,and high SDI countries were 99.37 (74.70–121.19), 93.46 (66.44–114.30), 68.96 (55.86–104.85), 32.79 (19.78–92.26) and 19.47 (18.53–22.32), respectively. In high-middle SDI region, age-standardized DALY rates and age-standardized prevalence rates were negatively correlated with SDI in Pearson correlation (r = − 0.234, *p* = 0.027) and (r = − 0.288, *p* = 0.006), with linear regression (equation: Y = − 333*X + 307.1) and (equation: Y = − 6187*X + 5588), respectively (Fig. 3C, D).

Wilcoxon Signed-Rank Test showed gender inequality in all SDI regions in terms of age-standardized DALY rates and age-standardized prevalence rates in 2019 (*p* < 0.001) (Fig. 3A, B). Further analyses revealed that female-minus-male difference in age-standardized rates of DALY and prevalence were both negatively related to SDI, in Pearson correlation (r = − 0.488, *p* < 0.001) and (r = − 0.437, *p* < 0.001), with linear regression (equation: Y = − 53.2*X + 50.0) and (equation: Y = − 492.8*X + 521.6), implying greater gender inequality in countries with lower SDI (Fig. 3E, F).

**Fig. 4:**
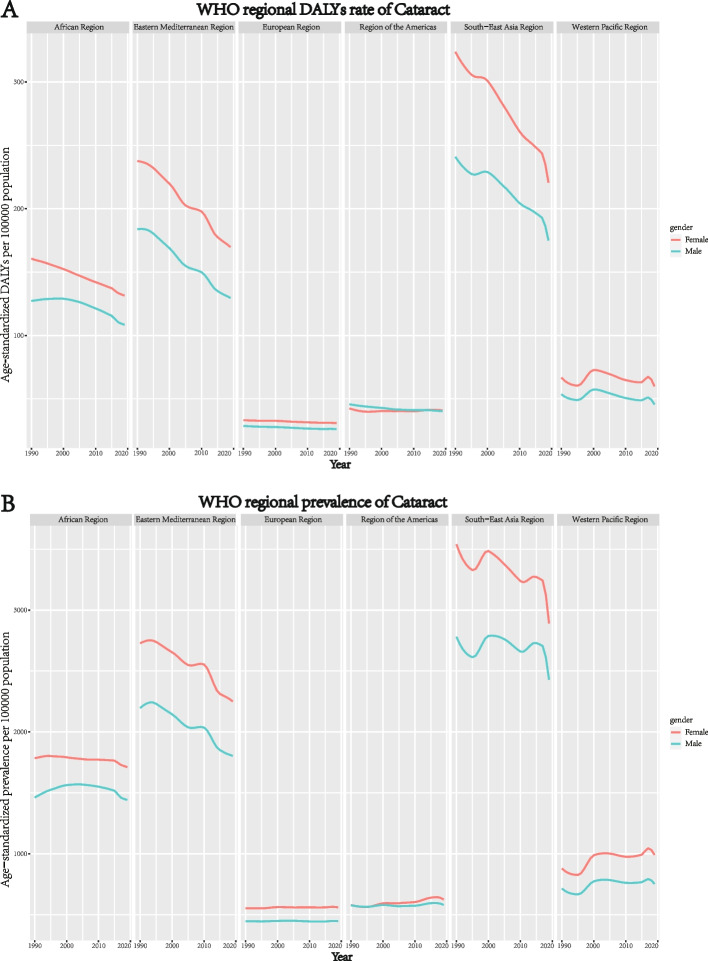
The persistence of gender inequality in WHO regional burden of cataracts in terms of age-standardized DALY rates (**A**), and age-standardized prevalence rates (**B**) from 1990 to 2019. DALYs = disability-adjusted life years

Gender inequality in the global burden of cataracts has persisted from 1990 to 2019 and has even gradually increased over the decades. The age-standardized DALY rates were 88.46 among males vs. 97.44 among females in 1990, and 74.91 vs.89.82 in 2019 (Figure S2). Regional trend plots revealed the persisting gender inequality in age-standardized DALY rates and prevalence rates in all WHO regions since 1990 through 2019 (Fig. 4). The gender inequality observed in the South-East Asia region was greater than that in the other WHO regions, with the highest both age-standardized rates of DALY and prevalence. In general, females had a higher burden of cataracts than males of the same time in all WHO regions, except in the African region.

### Cataract-related vision loss burden by GBD super regions

**Fig. 5:**
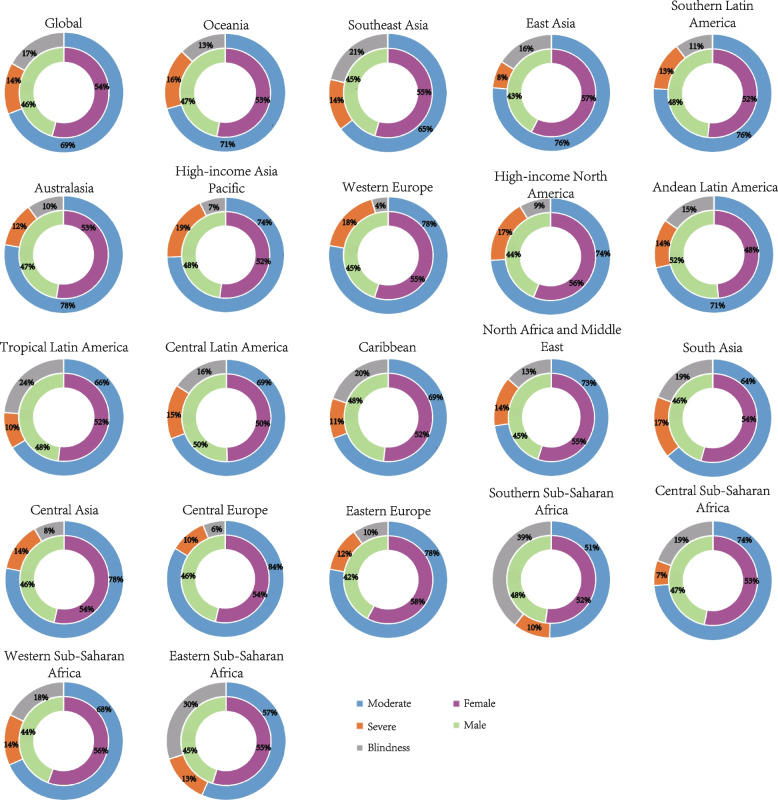
Age-standardized prevalence rates of blindness, moderate and severe vision impairment associated with cataracts by GBD super regions and gender in 2019. DALYs = disability-adjusted life years

The dual-pie charts depicted the proportion of gender and vision impairments burden due to cataract distribution in global and 21 GBD super regions in 2019 by adjusted prevalence rate (Fig. 3). In most GBD super regions, moderate vision loss took the majority parts, with Oceania [1740.26 (95% UI 1469.02–2026.46)], South Asia [1701.85 (95% UI 1417.98–2010.97)] and Southeast Asia [1692.06 (95% UI 1483.92–1899.73)] in top three places in terms of age-standardized prevalence rates. Meanwhile, in Southern Sub-Saharan Africa where blindness counted for the most [339.26 (95% UI 285.57–399.31)] by age-standardized DALY rates. In 19 out of 21 regions (besides the global situation), female subjects suffered a higher burden of cataract-related vision loss compared to males in terms of all vision loss. The most severe imbalance was observed in Eastern Europe (female versus male: 1.36 times) (Fig. 5).

**Fig. 6:**
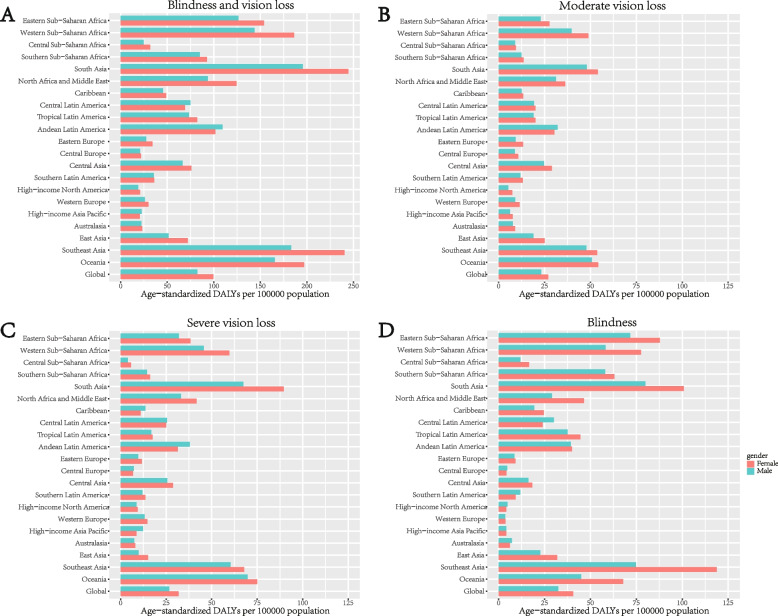
Age-standardized DALY rates of blindness, moderate, severe and all vision impairment associated with cataracts by GBD super regions and gender in 2019. DALYs = disability-adjusted life years

We then plotted DALYs data stratified by vision loss severity throughout GBD super regions (Fig. 4), showing blindness holds a great proportion. The leading three regions with the smallest DALYs rates were High-income North America [female 7.41 (95% UI 4.21–11.93) versus male 5.27 (95% UI 3.04–8.58)] in terms of moderate vision loss, Central Sub-Saharan Africa [female 5.66 (95% UI 3.37–8.78) versus male 3.94 (95% UI 2.36–6.26)] of severe and Western Europe [female 3.75 (95% UI 2.31–5.58) versus male 3.61 (95% UI 2.28–5.41)] of blindness. The adjusted DALYs rates of blindness and vision impairment in 2019 were slightly higher among females than among males across most of the regions (Fig. 6). Detail data on vision loss prevalence rate and DALYs rate due to cataract were displayed in Tables 1 and 2.

**Table 1:** Age-standardized DALYs rate (per 100,000 population) of vision loss due to cataract by GBD Super Region, 1990 and 2019, the Global Burden of Disease Study

GBD super region	1990											
	All vision loss			Moderate visual loss			Severe visual loss			Blindness		
	Male	Female	Both	Male	Female	Both	Male	Female	Both	Male	Female	Both
**Southeast Asia, East Asia, and Oceania**	108.27	140.22	126.06	22.21	25.57	24.02	23.75	27.31	25.64	62.31	87.35	76.40
Oceania	181.01	219.31	200.78	46.74	50.38	48.58	70.29	74.79	72.64	63.98	94.14	79.57
Southeast Asia	259.88	344.44	306.99	47.37	52.16	49.96	72.36	79.46	76.31	140.15	212.81	180.72
East Asia	59.37	78.04	70.50	14.51	17.69	16.37	8.16	11.55	10.21	36.71	48.80	43.92
**High-income**	24.76	27.09	26.29	7.32	9.36	8.63	11.71	12.24	12.06	5.73	5.49	5.59
Australasia	23.92	23.61	23.89	7.56	8.49	8.17	7.01	7.35	7.25	9.34	7.77	8.47
High-income Asia Pacific	24.39	22.39	23.28	5.93	7.24	6.76	12.19	8.79	10.20	6.26	6.36	6.33
Western Europe	27.32	31.03	29.69	8.92	11.14	10.38	13.56	14.90	14.38	4.84	4.99	4.92
High-income North America	18.73	21.57	20.52	5.08	7.20	6.40	8.81	9.84	9.46	4.83	4.53	4.67
Southern Latin America	42.14	42.39	42.50	11.23	12.47	12.02	12.62	14.58	13.80	18.29	15.34	16.69
**Central Europe, Eastern Europe, and Central Asia**	33.35	39.59	37.64	10.53	13.62	12.59	11.31	12.72	12.31	11.51	13.25	12.74
Central Asia	77.14	88.29	84.36	24.57	28.66	27.15	27.81	31.27	30.07	24.76	28.36	27.13
Central Europe	22.57	24.22	23.63	8.57	10.51	9.76	7.48	7.40	7.46	6.52	6.31	6.41
Eastern Europe	32.16	39.22	37.31	9.18	12.80	11.71	10.76	12.34	11.96	12.22	14.08	13.63
**Latin America and Caribbean**	103.32	103.56	103.60	19.73	20.03	19.88	24.86	23.24	24.00	58.74	60.29	59.72
Andean Latin America	157.49	148.77	153.06	33.63	32.18	32.87	42.83	34.51	38.53	81.03	82.08	81.66
Tropical Latin America	100.15	113.80	107.95	19.13	19.96	19.57	19.23	20.29	19.86	61.79	73.55	68.51
Central Latin America	107.62	94.55	100.87	19.36	19.53	19.45	29.07	27.62	28.31	59.20	47.40	53.11
Caribbean	58.51	64.66	61.82	12.16	12.92	12.55	14.63	11.62	13.04	31.73	40.12	36.23
**North Africa and Middle East**	132.65	181.77	157.51	29.48	35.15	32.30	37.18	49.45	43.28	65.99	97.17	81.93
**South Asia**	260.15	349.24	303.00	45.33	53.79	49.34	70.79	94.39	82.12	144.03	201.06	171.54
**Sub-Saharan Africa**	138.48	174.80	158.04	24.01	27.86	26.02	31.82	40.75	36.61	82.65	106.18	95.41
Southern Sub-Saharan Africa	124.88	136.72	131.93	11.81	13.20	12.59	15.46	16.40	16.04	97.61	107.13	103.30
Central Sub-Saharan Africa	30.71	40.95	36.27	7.78	8.23	8.04	3.79	5.29	4.60	19.13	27.43	23.63
Western Sub-Saharan Africa	154.52	205.45	181.53	32.08	38.63	35.40	40.44	57.21	49.27	82.00	109.62	96.85
Eastern Sub-Saharan Africa	150.90	190.57	171.60	22.59	27.03	24.86	34.37	41.78	38.22	93.93	121.75	108.52

GBD super region	2019											
	All vision loss			Moderate visual loss			Severe visual loss			Blindness		
	Male	Female	Both	Male	Female	Both	Male	Female	Both	Male	Female	Both
**Southeast Asia, East Asia, and Oceania**	80.15	110.02	96.71	25.15	31.51	28.61	20.72	26.90	24.14	34.28	51.60	43.96
Oceania	165.36	196.92	181.54	50.69	54.19	52.45	69.74	74.94	72.40	44.92	67.79	56.68
Southeast Asia	183.03	240.27	215.62	47.77	53.64	51.04	60.41	67.86	64.71	74.84	118.77	99.87
East Asia	51.51	71.94	63.32	18.97	25.03	22.33	9.91	15.06	12.88	22.63	31.86	28.11
**High-income**	23.32	25.24	24.48	7.40	9.45	8.60	11.47	11.63	11.59	4.44	4.16	4.29
Australasia	22.36	23.06	22.78	7.73	8.94	8.40	7.46	8.05	7.80	7.18	6.07	6.59
High-income Asia Pacific	22.55	20.45	21.43	6.19	7.62	7.03	12.26	8.70	10.28	4.10	4.13	4.12
Western Europe	25.82	29.80	28.15	9.04	11.39	10.42	13.17	14.66	14.04	3.61	3.75	3.69
High-income North America	18.74	20.85	19.98	5.27	7.41	6.48	8.69	9.33	9.06	4.78	4.12	4.43
Southern Latin America	35.54	35.94	35.92	11.80	13.11	12.62	12.00	13.56	12.93	11.73	9.27	10.37
**Central Europe, Eastern Europe, and Central Asia**	29.00	34.30	32.43	10.75	14.03	12.82	10.36	11.75	11.29	7.89	8.52	8.32
Central Asia	66.53	75.97	72.30	24.73	28.90	27.24	25.61	28.73	27.56	16.19	18.34	17.50
Central Europe	20.74	21.75	21.41	8.86	10.65	9.94	7.25	6.82	7.03	4.63	4.28	4.43
Eastern Europe	27.49	34.16	32.01	9.28	13.31	11.93	9.65	11.66	11.06	8.55	9.19	9.02
**Latin America and Caribbean**	75.05	75.66	75.54	19.86	20.38	20.13	22.21	21.18	21.65	32.99	34.10	33.76
Andean Latin America	109.34	101.69	105.48	32.10	30.37	31.18	37.94	31.35	34.50	39.31	39.97	39.80
Tropical Latin America	73.43	82.18	78.62	19.06	20.06	19.61	16.77	17.55	17.25	37.60	44.57	41.76
Central Latin America	74.84	68.97	71.72	19.26	20.03	19.67	25.49	25.03	25.24	30.09	23.91	26.81
Caribbean	45.51	48.94	47.41	12.50	13.39	12.97	13.65	10.97	12.20	19.35	24.58	22.24
**North Africa and Middle East**	93.48	124.40	108.81	31.28	36.29	33.75	33.18	41.67	37.35	29.02	46.45	37.71
**South Asia**	195.35	244.44	220.81	47.99	54.05	50.99	67.45	89.58	78.90	79.91	100.81	90.92
**Sub-Saharan Africa**	119.23	143.85	132.52	27.53	31.92	29.84	33.15	39.83	36.71	58.55	72.10	65.98
Southern Sub-Saharan Africa	84.97	92.68	89.75	12.45	13.54	13.07	14.44	16.11	15.45	58.08	63.02	61.22
Central Sub-Saharan Africa	24.71	31.60	28.73	8.91	9.37	9.17	3.94	5.66	4.95	11.85	16.56	14.61
Western Sub-Saharan Africa	143.71	186.12	165.99	39.73	48.85	44.52	45.70	59.74	53.07	58.28	77.52	68.40
Eastern Sub-Saharan Africa	126.36	153.77	141.04	22.82	27.65	25.36	31.92	38.34	35.33	71.63	87.78	80.35

**Table 2:** Age-standardized Prevalence rate (per 100,000 population) of vision loss due to cataract by GBD Super Region, 1990 and 2019, the Global Burden of Disease Study

GBD super region	1990											
	All vision loss			Moderate visual loss			Severe visual loss			Blindness		
	Male	Female	Both	Male	Female	Both	Male	Female	Both	Male	Female	Both
**Southeast Asia, East Asia, and Oceania**	1221.59	1496.69	1373.55	739.35	852.70	800.69	135.50	156.37	146.64	346.74	487.62	426.22
Oceania	2297.57	2629.18	2467.72	1548.90	1679.19	1614.75	395.32	424.92	410.72	353.34	525.07	442.25
Southeast Asia	2767.36	3386.32	3110.31	1575.84	1740.44	1664.78	411.57	455.46	436.07	779.96	1190.42	1009.46
East Asia	732.75	927.31	848.08	482.83	589.74	545.54	46.43	65.96	58.28	203.50	271.61	244.26
**High-income**	337.49	406.12	381.85	240.87	308.10	284.13	65.30	68.05	67.17	31.32	29.97	30.56
Australasia	340.31	363.76	356.57	249.85	280.16	269.62	39.24	41.04	40.55	51.22	42.56	46.40
High-income Asia Pacific	297.35	322.03	313.83	195.18	238.33	222.46	67.96	48.95	56.79	34.21	34.75	34.58
Western Europe	395.25	476.12	448.19	293.36	366.24	341.43	75.49	82.77	79.96	26.39	27.11	26.80
High-income North America	243.57	316.77	289.16	167.77	237.10	210.76	49.24	54.82	52.76	26.56	24.85	25.65
Southern Latin America	541.28	576.33	564.67	370.33	410.84	396.09	70.62	81.33	77.07	100.34	84.16	91.51
**Central Europe, Eastern Europe, and Central Asia**	475.97	595.93	556.50	348.74	450.99	416.73	63.61	71.61	69.30	63.63	73.33	70.46
Central Asia	1107.74	1283.61	1219.74	813.69	949.88	899.72	156.74	176.32	169.48	137.31	157.41	150.53
Central Europe	360.98	422.95	399.38	283.17	346.78	322.32	41.90	41.44	41.79	35.90	34.73	35.27
Eastern Europe	432.33	571.44	531.04	304.24	423.94	388.21	60.57	69.57	67.41	67.51	77.94	75.41
**Latin America and Caribbean**	1122.22	1132.29	1128.17	655.30	665.01	660.20	140.85	131.76	136.00	326.07	335.52	331.97
Andean Latin America	1809.63	1722.47	1764.55	1116.70	1068.69	1091.42	242.65	195.91	218.46	450.28	457.87	454.68
Tropical Latin America	1087.52	1187.21	1143.56	635.27	662.95	649.98	109.01	115.12	112.66	343.24	409.14	380.93
Central Latin America	1136.36	1069.67	1101.92	643.20	648.88	646.17	164.66	156.52	160.39	328.50	264.27	295.36
Caribbean	661.41	715.59	690.17	403.06	428.02	416.00	82.61	65.61	73.65	175.74	221.96	200.52
**North Africa and Middle East**	1554.70	1993.37	1775.75	977.86	1169.61	1073.53	210.13	280.60	245.18	366.71	543.16	457.04
**South Asia**	2734.89	3494.36	3098.49	1517.92	1808.37	1655.57	406.17	545.79	473.20	810.79	1140.21	969.72
**Sub-Saharan Africa**	1439.37	1751.23	1605.55	798.86	927.34	865.83	180.62	231.80	208.10	459.89	592.08	531.62
Southern Sub-Saharan Africa	1021.92	1126.35	1082.82	393.45	439.48	419.40	87.51	92.83	90.83	540.95	594.04	572.60
Central Sub-Saharan Africa	387.16	457.23	425.58	259.15	274.15	267.71	21.58	30.07	26.15	106.43	153.00	131.71
Western Sub-Saharan Africa	1752.52	2220.28	1996.56	1066.05	1283.69	1176.49	229.39	324.79	279.72	457.08	611.79	540.35
Eastern Sub-Saharan Africa	1470.36	1819.96	1651.44	752.89	901.86	828.90	195.41	238.53	217.84	522.06	679.56	604.70

GBD super region	2019											
	All vision loss			Moderate visual loss			Severe visual loss			Blindness		
	Male	Female	Both	Male	Female	Both	Male	Female	Both	Male	Female	Both
**Southeast Asia, East Asia, and Oceania**	1138.48	1482.83	1327.84	832.36	1044.48	947.86	116.82	152.26	136.49	189.30	286.08	243.50
Oceania	2315.37	2605.10	2463.23	1676.24	1802.96	1740.26	391.25	424.59	408.31	247.87	377.54	314.66
Southeast Asia	2337.53	2825.38	2613.62	1581.87	1779.53	1692.06	340.91	385.08	366.42	414.75	660.77	555.14
East Asia	808.10	1090.75	967.90	627.66	829.41	739.72	55.80	85.09	72.77	124.64	176.26	155.41
**High-income**	331.62	397.74	370.41	243.53	310.47	282.59	63.81	64.59	64.38	24.28	22.69	23.44
Australasia	335.48	371.47	355.89	254.73	293.55	276.53	41.55	44.82	43.41	39.20	33.11	35.94
High-income Asia Pacific	294.05	320.98	310.34	203.60	250.25	230.82	68.09	48.25	57.06	22.37	22.48	22.46
Western Europe	389.66	475.34	440.10	296.88	373.67	342.14	73.11	81.30	77.89	19.67	20.38	20.06
High-income North America	248.66	318.48	288.60	173.81	243.87	213.67	48.54	51.98	50.57	26.31	22.63	24.35
Southern Latin America	520.42	556.85	544.05	389.06	430.69	415.20	67.01	75.33	71.98	64.34	50.84	56.87
**Central Europe, Eastern Europe, and Central Asia**	456.76	576.69	532.95	355.32	463.76	423.75	58.03	65.95	63.36	43.40	46.98	45.84
Central Asia	1051.44	1218.98	1152.73	818.00	955.89	900.94	143.96	161.56	154.95	89.49	101.54	96.83
Central Europe	358.06	412.79	391.55	292.18	351.16	327.95	40.47	38.12	39.26	25.40	23.51	24.34
Eastern Europe	408.45	556.85	506.85	307.11	440.45	394.79	54.18	65.68	62.25	47.15	50.73	49.80
**Latin America and Caribbean**	964.47	982.17	974.73	657.14	674.08	666.08	125.11	119.31	121.94	182.22	188.78	186.70
Andean Latin America	1494.22	1403.73	1447.36	1062.70	1004.97	1032.11	213.86	176.85	194.51	217.66	221.91	220.74
Tropical Latin America	931.80	1008.10	975.80	630.03	663.02	648.07	94.26	98.77	97.01	207.51	246.32	230.71
Central Latin America	947.92	936.81	942.18	637.93	662.99	651.43	143.67	141.07	142.26	166.32	132.75	148.49
Caribbean	597.46	639.75	620.33	413.81	442.50	429.02	76.81	61.67	68.62	106.84	135.57	122.68
**North Africa and Middle East**	1379.67	1691.70	1534.09	1032.97	1200.01	1115.46	186.18	234.06	209.70	160.52	257.64	208.94
**South Asia**	2430.63	2887.60	2663.74	1599.15	1806.21	1701.85	383.76	512.93	450.60	447.72	568.47	511.30
**Sub-Saharan Africa**	1426.96	1684.12	1563.89	914.06	1058.56	989.78	187.62	225.09	207.61	325.28	400.47	366.50
Southern Sub-Saharan Africa	817.44	889.83	860.85	413.84	449.73	434.35	81.55	90.98	87.24	322.05	349.12	339.26
Central Sub-Saharan Africa	384.45	435.09	413.80	296.38	311.35	304.81	22.31	32.02	28.04	65.75	91.72	80.95
Western Sub-Saharan Africa	1900.12	2386.00	2155.19	1317.62	1617.93	1475.13	258.38	337.21	299.77	324.13	430.86	380.29
Eastern Sub-Saharan Africa	1336.72	1624.11	1489.44	758.38	918.89	842.86	180.81	217.38	200.28	397.54	487.84	446.30

### Predicted global burden of cataract

ARIMA model was used to predict the global burden of cataracts in terms of age-standardized rates of DALY and prevalence beyond 2019. Generally, both changes in age-standardized DALY rates and prevalence rates were in the wave trend, they declined from 1990 to 1995 and then increase, but there was a significant decrease since 2017. Nevertheless, age-standardized DALYs and prevalence rates of cataracts peaked in 2000 and 2017, with 94.52 (95% UI 67.09–127.24) per 100,000 population and 1283.53 [95% uncertainty interval (UI) 1134.46–1442.93], respectively. Age-standardized DALY rates showed a trend of fluctuation by ARIMA (2,0,2) model beyond 2019, estimated as 91.52 (95% UI: 87.11–95.94) by 2050. While another ARIMA (3,1,0) model revealed that age-standardized prevalence rates would keep steady at 1232.33 (95% UI 942.33–1522.33) by 2050 (Fig. S3).

## Discussion

This study comprehensively demonstrated the trend of the global burden of cataracts by year, age, gender, and socioeconomic status with measurements of prevalence and DALYs. We found that gender inequality in the global burden of cataracts had persisted since 1990, increased with age, and was greater in countries and territories with lower socioeconomic development. From 1990 to 2019, global DALY numbers and crude rates rose gradually, while age-standardized rates declined. Since age-standardized prevalence rates exclude the effect of population size and age structure, revealing the true burden of disease. It indicates that progress was made in the fight against cataracts, but we still face enormous challenges in avoiding vision impairment caused by cataracts, due to the growing and aging population.

In an era of shifting global agendas, more emphasis is expanded on non-communicable diseases and injuries along with communicable diseases. Ono et al. reported for the first time that cataracts have the most uneven distribution at the global level among non-communicable eye diseases, using disability-adjusted life-year data from the 2004 GBD study [20]. Yan and his colleagues [21] based on the data from the GBD 2017 study, found socioeconomic status was inversely associated with the burden of cataracts, which has been also confirmed in our study [22]. A similar trend has been visible in some previous investigations [22, 23]. HDI (human development index) was mostly used in previous studies, which is a product published by the United Nations Development Programme (UNDP) covering social and economic factors in three areas (health, education, and living standards) [14]. However, changes were made to the definition of HDI before and after 2010. To ensure comparability between different years, the development status of a country/region was evaluated by SDI in our study. We found that age-standardized DALY rates differed among countries with different SDI regions, with cataract burden being more concentrated in countries with lower socioeconomic status. In high-middle SDI countries, a significant negative correlation exists between age-standardized DALY rates and SDI. Similarly, socioeconomic disparity also exists in many eye diseases, Li et al. found less developed countries tend to have a higher burden of uncorrected refractive error [24].

This association may be due to better eye care and greater access to cataract surgery in countries with high SDI. The research found that the prevalence of blindness was negatively correlated with the density of ophthalmologists. Higher national income was associated with a higher density of ophthalmologists, ranging from an average of 76.2 ophthalmologists per million population in high-income countries to an average of 3.7 ophthalmologists in low-income countries, with a difference of 18 times [15]. Wang eal. Documented the strong associations of socioeconomic indices with quantity and quality of cataract surgery, the countries with the highest socioeconomic level had the best cataract surgery outcomes [14]. Low-income countries with lower cataract surgical coverage, in part due to inadequate training opportunities for young surgeons [25].

In 1999, the WHO and the International Agency for the Prevention of Blindness (IAPB) launched the VISION 2020 global initiative to eliminate avoidable blindness by 2020 [26]. Cataracts and uncorrelated refractive errors are considered the main objects of WHO and VISION 2020 initiatives. However, the refractive error can be corrected by spectacles, there is no effective conservative method for cataracts except surgery. Cataract is the leading global cause of blindness in those aged 50 years and older [27], impairs vision, and affects the patient’s quality of life. Improving vision timely facilitates many daily life activities, enables better educational achievements, and increases work productivity, reducing inequality. Studies have also shown that cataract surgery can reduce the risk of dementia and fall. The incidence of falls among older people referred for cataract surgery was 31% lower after first eye surgery, and a further 50% lower after second eye surgery, by restoring binocular vision [9]. Cataract surgery is a disability-preventing and highly cost-effective intervention costing less than $100 per DALY avoided [28]. Therefore, cataract surgery leads to huge socioeconomic benefits and improvement in well-being and quality of life. Nonetheless, cataract surgery is not a panacea for all problems. With the population aging and life expectancy rising, demand for cataract surgery is surging. A total of $5733 million investment was estimated to be required for eliminating blindness due to cataracts between 2010 and 2020, which is a great financial burden and a challenge to public health [29]. Beyond that, the incidence of cataract surgical complications was 1.2%, including posterior capsular rupture, cystoid macular edema, retinal detachment, and endophthalmitis [30]. Most of these complications of surgery may need further intra- or postoperative management. Although DALY is lower in high-income countries, it may require higher environmental costs, against the idea of sustainable development. For example, a phacoemulsification cataract extraction in a UK hospital produced more than 20 times the greenhouse gas emission of the same procedure in an Indian hospital [31]. Progress in improving the global burden of cataracts has been achieved in recent years, but much more remains to be done.

Previous studies mostly concentrated on the overall visual impairment caused by cataracts, but the different levels of visual impairment (moderate, severe vision loss, and blindness) remain unknown. We found moderate vision loss took the majority in terms of age-standardized prevalence rates of cataracts, while age-standardized DALY rates of blindness predominated. DALY reflects the gap between the actual health status and the normative situation. It indicates that cataract blindness damages the healthy life years of patients and quality of life seriously, even at low prevalence rates. What’s more, we found gender inequality in the burden of cataracts had persisted since 1990, and the inequality remained with aging among different stages of vision impairments and regions with different levels of development. Other studies have found similar patterns of gender disparity [23], including but not limited to cataracts, uncorrected refractive error [24], age-related macular degeneration [32], and diabetes retinopathy [33] also share the same pattern.

One possible explanation is that women have a higher prevalence of cataracts and a longer life expectancy. According to the World Health Organization’s official website, the average life expectancy of women in 2020 is generally higher than that of men. Japan ranks first with an average life expectancy of 83.7 years, while the average life expectancy of women is 86.8, while that of men is only 80.5 [34]. Another explanation may have to do with gender inequality, which has been a problem throughout the world for many years. There is evidence suggesting that women are disadvantaged in areas such as education, job opportunities, income distribution, and medical care [35]. Although 60% of cataract patients are women, men are 1.39 times more likely than women to undergo cataract surgery [36]. Compared with men, women have less support from family and less control over finances, which may hinder their access to cataract surgery. For children with bilateral cataracts, girls are also less likely to undergo surgery than boys in low-income countries [37]. Therefore, more attention should be paid to eye care services for women, eliminating gender inequality is an important part of combating the global burden of cataracts.

The limitations of this study should not be ignored. First, the accuracy of health burden information is limited by the source of original data, which the GBD 2019 study noted in their reports [17]. In absence of data, the out-of-sample prediction validity dependent on modeling is bound to bring some deviation. Second, the use of aggregate data at the first level of administrative organization within each country level ignoring data from subnational locations would be a source of bias. There can be great variation inside a country, and extending conclusions to a particular region should be cautious. Moreover, COVID-19 has changed the way people live around the world, which may lead to a decrease in the predictive accuracy of ARIMA models. In the outpatient clinic, due to the lockdown policy, we found that many cataract patients have poor vision when they see a doctor, and the times waiting for cataract surgery are longer, which is bound to affect their quality of life.

In summary, this study showed global health progress in cataracts with age-standardized DALYs rate decreasing in the past few decades. The aging of the population is outpacing the growth of the profession, cataract still reminds a global public health concern. Cataract-related health services should be strengthened for the older population, females, and people in lower SDI regions. Hopefully, our study could raise awareness of the disease burden of cataracts and would be valuable for policy making and program planning.

## Supplementary Information

**Additional file 1: Figure S1-3.**

## Data Availability

Data was acquired from the Global Health Data Exchange (http://ghdx.healthdata.org/gbd-results-tool).

## Abbreviations

DALY: Disability adjusted life years
GBD: Global Burden of Disease
WHO: World Health Orgnization
YLD: Years lived with disability
YLL: Years of life lost
SDI: Socio-demographic Index
MSVI: Moderate and serve visual acuity
VA: Visual acuity
ARIMA: Auto- Regressive Integrated Moving Average
UI: Uncertainty intervals
HDI: Human development index
UNDP: United Nations Development Programme
