# Supplementary material for: Global, regional, national burden and gender disparity of cataract: findings from the global burden of disease study 2019
Source: BMC Public Health. 2022 Nov 12;22:2068. doi: 10.1186/s12889-022-14491-0 (PMC9652134; doi:10.1186/s12889-022-14491-0)
Supplement: Supplementary file 1 — Additional file 1: Figure S1-3. [file 12889_2022_14491_MOESM1_ESM.pdf]

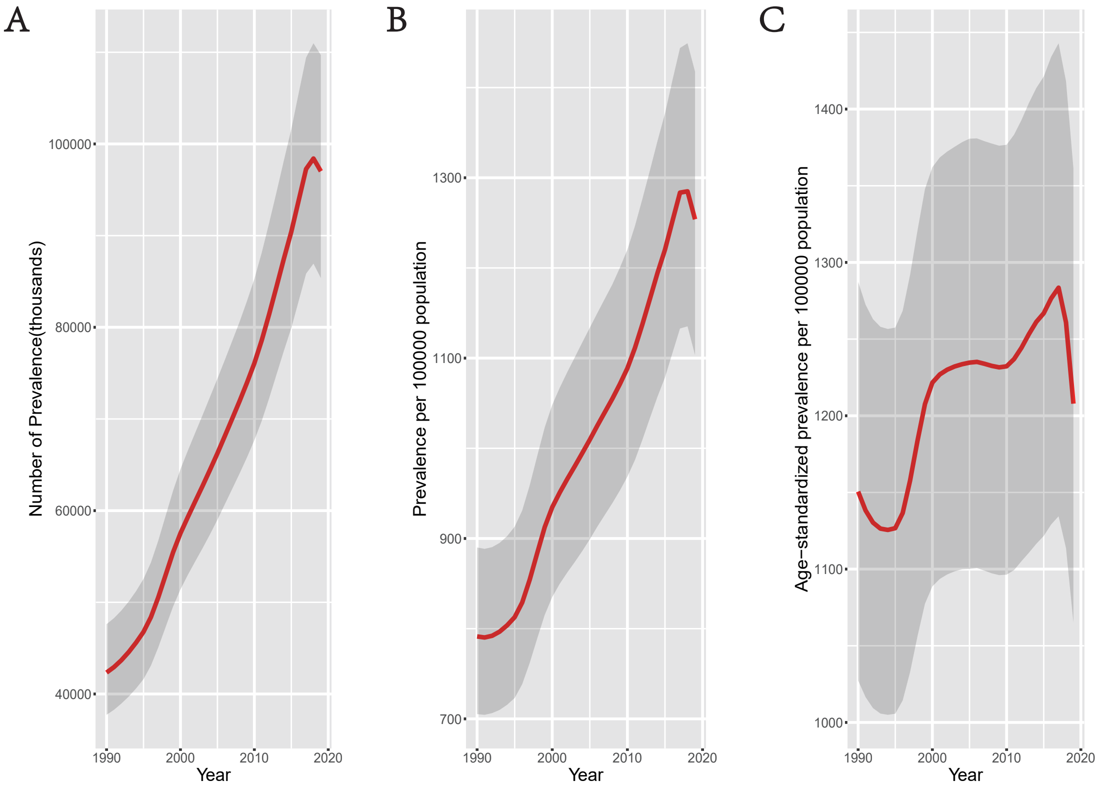

A
Number of Prevalence(thousands)
100000
80000
60000
40000
1990
2000
2010
2020
Year
B
Prevalence per 100000 population
1300
1100
900
700
1990
2000
2010
2020
Year
C
Age−standardized prevalence per 100000 population
1400
1300
1200
1100
1000
1990
2000
2010
2020
Year

FigureS1

Trends in global burden of cataracts in terms of prevalence numbers (A), crude prevalence rates (B), and age-standardized prevalence rates (C), from 1990 to 2019. Shaded areas represent 95% uncertainty intervals. DALYs = disability-adjusted life years

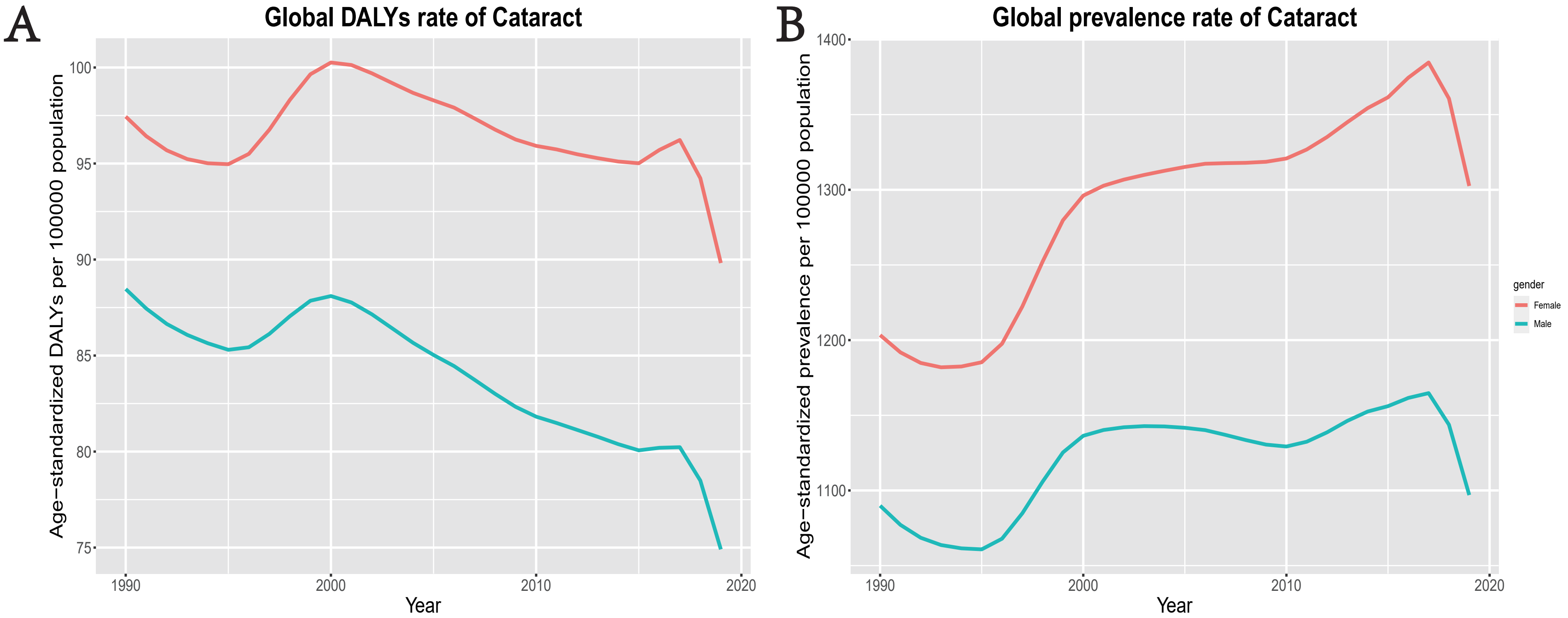

A
Global DALYs rate of Cataract
Age−standardized DALYs per 100000 population
100
95
90
85
80
75
1990
2000
2010
2020
Year
B
Global prevalence rate of Cataract
Age−standardized prevalence per 100000 population
1400
1300
1200
1100
1990
2000
2010
2020
Year
gender
Female
Male

FigureS2

The persistence of gender inequality in global burden of cataracts from 1990 to 2019, in terms of age-standardized DALY rates (A), and age-standardized prevalence rates (B). DALYs=disability-adjusted life years.

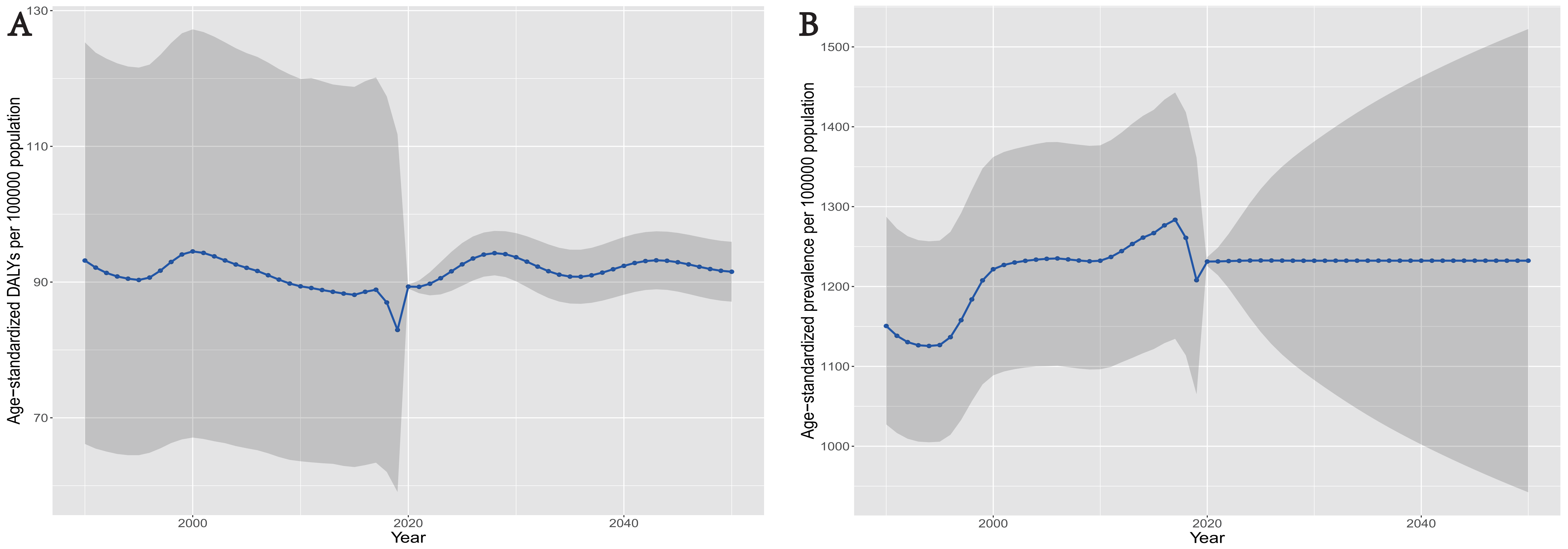

A
Age-standardized DALYs per 100000 population
130
110
90
70
2000
2020
2040
Year
B
Age-standardized prevalence per 100000 population
1500
1400
1300
1200
1100
1000
2000
2020
2040
Year

FigureS3

Global burden of cataracts from 1990 to 2050 in terms of age-standardized DALY rates (A), and age-standardized prevalence rates (B). Shaded areas represent 95% uncertainty intervals. DALYs = disability-adjusted life years.
